# Percutaneous Stabilization System Osseofix® for Treatment of Osteoporotic Vertebral Compression Fractures - Clinical and Radiological Results after 12 Months

**DOI:** 10.1371/journal.pone.0065119

**Published:** 2013-06-26

**Authors:** Stephan Albrecht Ender, Elmar Wetterau, Michaela Ender, Jens-Peter Kühn, Harry Rudolf Merk, Ralph Kayser

**Affiliations:** 1 Department of Orthopaedics and Orthopaedic Surgery, University Medicine Greifswald, Greifswald, Germany; 2 Department of Diagnostic Radiology and Neuroradiology, University Medicine Greifswald, Greifswald, Germany; The James Cook University Hospital, United Kingdom

## Abstract

**Study Design:**

A prospective consecutive cohort study (follow-up study).

**Objective:**

Our study investigated whether implantation of an expandable titanium mesh cage (Osseofix®) is a successful and safe minimally invasive therapy for osteoporotic vertebral compression fractures (VCF). Our experiences, clinical and radiological findings after 12 months follow-up are presented. Kypho- and vertebroplasty are well-established minimally invasive procedures for the treatment of osteoporotic VCF. The main complications associated with both procedures are uncontrolled bone cement leakage. Therefore a suitable alternative has been investigated.

**Methods:**

During June 2010 to May 2011 24 patients were included with 32 osteoporotic VCF (T6 to L4). All of them were stabilized with the Osseofix® system. Preinterventionally we performed X-ray, MRI, and bone density measurements (DXA). Clinical and radiological results were evaluated preop., postop. and after 12 months postop. based on the Oswestry Disability Index (ODI) and the Visual Analogue Scale (VAS), X-ray (Beck Index, Cobb-angle) and CT.

**Results:**

There was a significant improvement in the mean ODI (70,6% to 30,1%) as well as a significant reduction in pain intensity (VAS) (7,7 to 1,4) after 12 month. The mean kyphotic angle according to Cobb showed significant improvements (11,7° to 10,4°) after 12 months. Postinterventional imaging showed only one case of loss of height in a stabilized vertebral body (3.1%). We saw no changes in posterior vertebral wall or adjacent fractures. Except for one pronounced postoperative hematoma we saw no surgical complications including no cement leakage.

**Conclusions:**

Stabilization of symptomatic osteoporotic VCF with Osseofix® system is a safe and effective procedure, even in fractures with posterior wall involvement. The clinical mid-term results are good at a very low complication rate. The Osseofix® system is an interesting alternative to the established procedures of cement augmentation.

## Introduction

Due to the demographic changes in Western Europe [1] [2] and North America [3] the number of patients suffering from osteoporosis and osteoporotic vertebral fractures are expected to increase [2] [4]–[7]. The German Umbrella Organization of Osteology (DVO - Dachverband Osteologie e. V.) published clear recommendations for the management of stable, recent, symptomatic osteoporotic vertebral fractures in patients without neurological deficits [8]: Patients should be mobilized as quickly as possible to avoid potential complications of immobility. Conservative treatment, which should be attempted first, includes adequate pain management and possibly spinal orthosis [8]. Specific medical osteoporosis treatment is indicated for isolated osteoporotic vertebral fracture grade 2 or 3 according to Genant [9] (25–40% or >40% loss of height) or multiple vertebral fractures Genant grade 1 to 3 (20%–>40% loss of height) that are accompanied by T-values of ≤−2.0 (DXA) [8]. In case of failed conservative treatment with insufficient pain relief after three weeks, surgical stabilization of the spine (vertebro- or kyphoplasty) should be considered [8]. These procedures have proven to be good alternatives to non-surgical treatment [10]–[12] for both osteoporotic and tumor-related vertebral fractures [10] [13]. Specific complications of these two procedures are mainly due to accidental leakage of bone cement [10] [13].

Since 2009, expandable titanium mesh cages (Osseofix® vertebral fracture repositioning system) are available. They represent a new percutaneous stabilization method of osteoporotic throracolumbar vertebral compression fractures [14] [15].

Our study investigated whether the implantation of expandable titanium mesh cages (Osseofix®) is a suitable minimally invasive procedure for stabilizing osteoporotic vertebral compression fractures. We paid attention to the following parameters: operation time, X-ray exposure, pain reduction, functional outcome, sagittal alignment and complications. This paper presents our experiences with the Osseofix® system in a cohort of 24 patients with 32 vertebral compression fractures at 12 month follow-up.

## Materials and Methods

### Ethics statement

The study was approved by the Ethics Committee at the University Medicine Greifswald (reference number BB 132/12).

Patients were informed about all procedures and also asked to participate in the study. Only those who gave their verbal informed consent were included and their verbal consent was documented in their patient charts.

The Ethics Committee agreed on this procedure.

### Patients

This prospective follow-up study included 24 patients suffering from single or multiple symptomatic vertebral compression fractures (AO-types A1.1 to A1.3 and A3.1). In 8 cases MRI showed involvement of the posterior vertebral wall (AO-types A3.1). After a non-surgical attempt all subjects were treated with expandable titanium mesh cages (Osseofix®) (Alphatec Spine Inc., Carlsbad, California, USA) from June 2010 to May 2011.

We report on 20 female patients and 4 male patients. The average age at surgery was 67 years (range: 55 to 89 years). The average duration of symptoms was 8.7 weeks (range: 3 to 15 weeks). A total of 18 lumbar vertebrae were stabilized (6 L1, 4 L2, 5 L3, 3 L4), along with 14 thoracic vertebrae (1 T6, 3 T8, 2 T9, 1 T10, 3 T11, and 4 T12). In 17 patients in one session one fractured vertebra was stabilized, in 6 patients in one session 2 vertebrae were stabilized, and in one patient in one session 3 vertebrae were stabilized. In one patient both a T12 fracture and a L1 fracture were stabilized. At lumbar spine a bipedicular approach was used. The lower thoracic spine (T9–12) was stabilized with bipedicular implants as well. From T8 upwards, lateral extrapedicular approach to place the titanium mesh was performed.

In case of a non-injured vertebra between two fractured vertebrae, the non-injured vertebra was not stabilized.

***Inclusion criteria*** were persistent, symptomatic, recent lumbar or thoracic osteoporotic vertebral fractures (proven by X-ray and MRI) and unsatisfactory pain relief after at least 3 weeks of conservative treatment, according to DVO guidelines [8].

***Exclusion criteria*** were neurological deficits, due to posterior vertebral wall involvement that caused relevant spinal canal stenosis and known allergies to the contents of the Osseofix® systems or to bone cement.

Preoperative diagnostics included standard clinical examination and evaluation (Oswestry Disability Index (ODI) [16] and the Visual Analogue Scale (VAS)), X-ray of relevant spinal region in two views, MRI (T1- and T2-weighted sequences (sagittal and transversal) including fat suppression sequences (sagittal) and bone density measurement (DXA).

Clinical and radiological follow-up was performed three days after surgery and 12 months postoperatively (range: 12 to 14 months) in our spine outpatient clinic.

***Clinical follow-up*** evaluation was based on the ODI and VAS. Additional the Smiley-Webster scale was used after 12 month to classify the parameter patient satisfaction, based on pain status, as “excellent,” “good,” “fair,” or “poor” [17].

***Radiological follow-up*** included X-ray in two views, standing position, and postoperatively CT.

**Figure 1:**
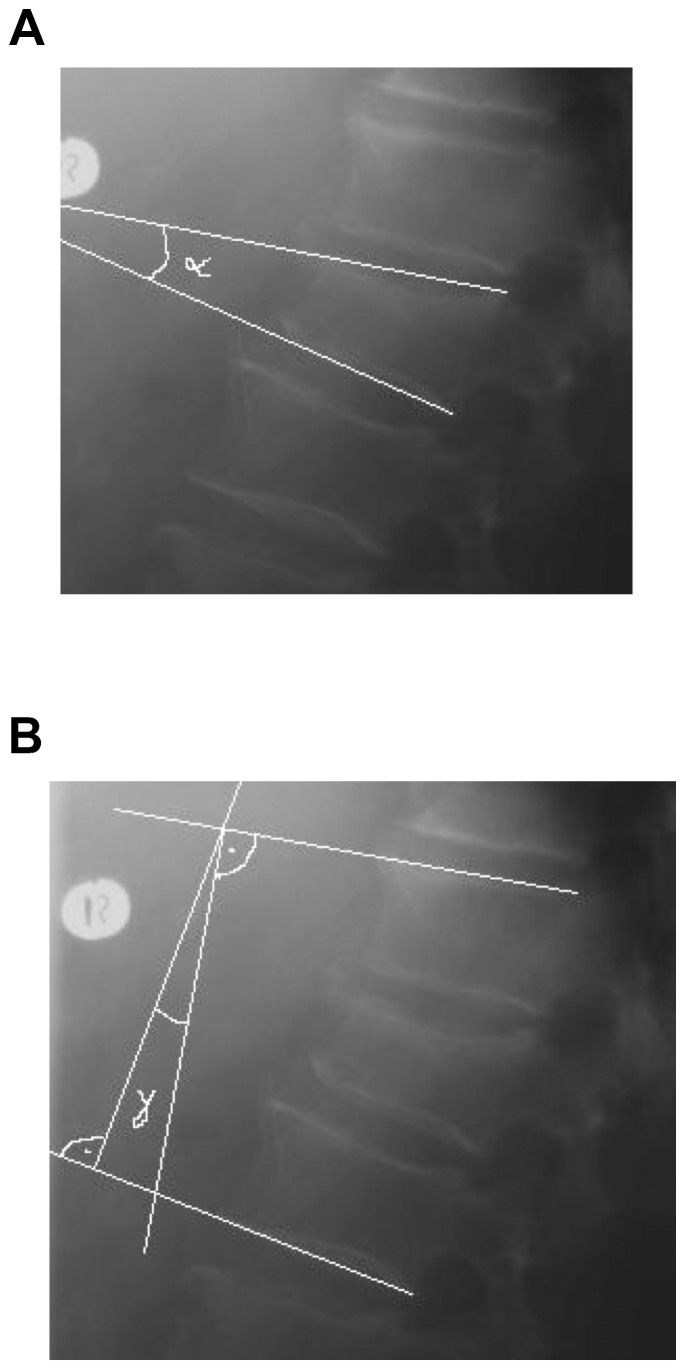
Radiological angle determination. **(a and b): Radiological follow up evaluation. Determining the α- and γ-kyphotic angles.**

Quantitative evaluation of spinal deformity was based on the Beck index [18] (anterior vertebral height/posterior vertebral height), kyphotic angle (α-angle), and regional kyphotic angle (γ-angle) according to Cobb [19] (Figure 1). In patients with non-adjacent vertebral fractures the regional kyphotic angle was determined separately for each level of the spine. In patients with neighboring vertebrae fractures the regional kyphotic angle was determined over all affected vertebral bodies. By definition in kyphotic deformity the angle has a positive value, and in lordosis it has a negative value [20].

The radiological follow up included an evaluation of the stabilized vertebra in terms of loss of height, cement exudation and changes to the posterior vertebral wall. It also included evaluation of neighboring vertebrae for potential adjacent fractures.

We analysed the correlation between ODI and changes in sagittal alignment.

### Surgical technique/Application of the Osseofix® system

**Figure 2:**
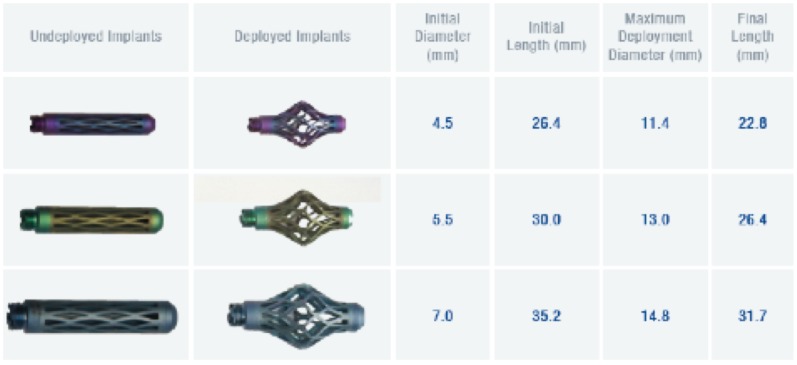
Osseofix® implants. Osseofix® for treatment of osteoporotic vertebral compression fractures. Osseofix® implants with technical specifications [Alphatec Spine Inc., Carlsbad, California, USA].

The Osseofix® system was used to treat all patients included in this study. Three implant sizes are available with diameters (non-expanded) of 4.5 mm, 5.5 mm, and 7.0 mm (Figure 2) which are intended for T1 to L5 respectively. The implant consists of a combination of titanium alloy (Ti-6Al-4V, ASTM F 136) and pure titanium (Ti-CP2, ASTM F67). The Osseofix®-implant size was chosen based on the pedicle dimensions and the vertebral body dimensions.

The average operation time per vertebra was 55 minutes (SD ±7.9, range: 35–91 minutes). The average X-ray exposure time was 1.37 minutes (SD ±0.27, range: 0.45–2.89 minutes) with a cumulative radiation dosage of 19.8 milliGray (SD ±1.9, range: 7.4–35.2 milliGray).

In 30 vertebrae (22 patients) the Osseofix® implant with smallest size (4.5 mm not expanded) was applied from T6 to L4. In 2 vertebrae (2 patients) the 5.5 mm diameter (L1–2) was used. For bipedicular implants always the same size was used.

In every case the surgery was performed under general anesthesia and the patients received a perioperative intravenous single shot antibiotic prophylaxis (1.5 g cefuroxime).

The surgical procedure was standardized as described for the Osseofix® system [9], [10]. The Osseofix® system is currently approved only using bone cement.

An average of 0.6 ml cement was used for each titanium mesh (SD ±0.11, range: 0.5–0.9 ml), with larger implants demanding more cement.

After vertebral stabilization patients were mobilized as quickly as possible. Standing at the first postoperative day was initiated under physiotherapeutic guidance. Further physiotherapy was also performed to strengthen the spine-stabilizing muscles. Analgesics were continued after surgery but gradually reduced. Oral bisphosphonate therapy was initiated or continued after surgery.

***Statistical analysis*** was performed using SAS®-Version 9.1 with the Wilcoxon rank-sum test for continuous variables (α- and γ-kyphotic angle) and the sign test for discontinuous variables (ODI and VAS). Evaluation for an association between postoperative kyphotic angle according to Cobb and clinical results was based on Fisher's exact test. The results were summarized with mean values and standard deviations (SD). P<0.05 was considered significant for disproving the null hypothesis.

## Results

### Clinical evaluation

24 patients (32 vertebral fractures) were evaluated preoperative, three days and 12 months after surgery.

The average preoperative pain value based on VAS was 7.7 points (SD ±1.8; range: 5–10 points). The average postoperative VAS was 1.7 points (SD ±0.89; range: 0–3 points). At follow-up (12 months later) the average VAS was 1.4 points (SD ±0.98; range: 0–3 points).

The average preoperative ODI was 70.6% (SD ±4.2, range: 62–78%). The average postoperative ODI was 30.6% (SD ±4.4; range: 24–48%). At follow up the average ODI was 30.1% (SD ±3.5; range: 24–42%) (Table 1).

**Table 1:** Changes in ODI and VAS.

Clinical evaluation	Average value preop.	Average value 3 days postop.	Average value after 12 month	Average change preop.-12 month	p-value preop.-12 month
**ODI**	**70.6%**	30.6%	**30.1%**	−40.5	p<0.001
**VAS**	**7.7**	1.7	**1.4**	−6.3	p<0.001

(32 fractures in 24 patients) – preoperative, 3 days postoperative and at 12-month follow-up.

According to the Smiley-Webster scale in 23 of 24 patients the clinical result was excellent or good (96%). In one case the result was fair. There were no poor results (Table 2).

**Table 2:** Smiley-Webster scale (patient satisfaction).

Patient satisfaction	Pain status after 12 month	Number (Fractures/Patients) n = 32/24
Excellent	No pain	25/19
Good	Rare or mild pain, no analgesics required	6/4
Fair	Moderate pain, analgesics occasionally required	1/1
Poor	Severe or lasting pain, analgesics regularly required	0/0

Results of treatment after 12-month follow-up to classify the parameter patient satisfaction, based on pain status (Smiley-Webster scale).

Highly significant improvements (p<0.001) in the average ODI and VAS were found after 12 months (ODI change from 70.6% to 30.1% and VAS change from 7.7 to 1.4 points).

### Radiological evaluation

Vertebral body deformation, measured using the Beck index, only changed minimally when comparing the preoperative condition (0.75 (SD ±0.16, range: 0.42–1.1)) with the condition after 12 months (0.78 (SD ±0.16, range: 0.5–1.1)). The Beck index did not change from day of discharge to the follow-up date.

We noticed a change in the average vertebral kyphotic angle (α-angle) when comparing the preoperative angle (9.0° (SD ±6.5; range: −2.8–21.9)) to the postoperative angle (8.0° (SD ±6.1; range: −2.8–20.2)) and the angle at follow-up (8.1° (SD ±6.0, range: −2.8–20.1)). We saw improvements in the kyphotic angle according to Cobb (γ-angle) when comparing the preoperative angle (11.7° (SD ±17.6, range: −38–34)) to the postoperative angle (10.4° (SD ±17.8, range: −44–33)) and the angle at follow-up (10.4° (SD ±17.6, range: −43–35)) (Table 3).

**Table 3:** Changes in sagittal spine alignment.

Sagittal spine alignment	Average value preop.	Average value 3 days postop.	Average value after 12 month	Average change preop.- 12 month	p-value preop.-12 month
**vertebral kyphotic angle** (α-angle)	**9.0°**	8.0°	**8.1°**	−1.0	p<0.05
**kyphotic angle accord. to Cobb** (γ-angle)	**11.7°**	10.4°	**10.4°**	−1.3	p<0.05

(32 fractures in 24 patients) – preoperative, 3 days postoperative and after 12-month follow-up.

In none of the stabilized vertebrae changes in the posterior vertebral wall (fragment dislocation) and or cement leakage was found.

**Figure 3:**
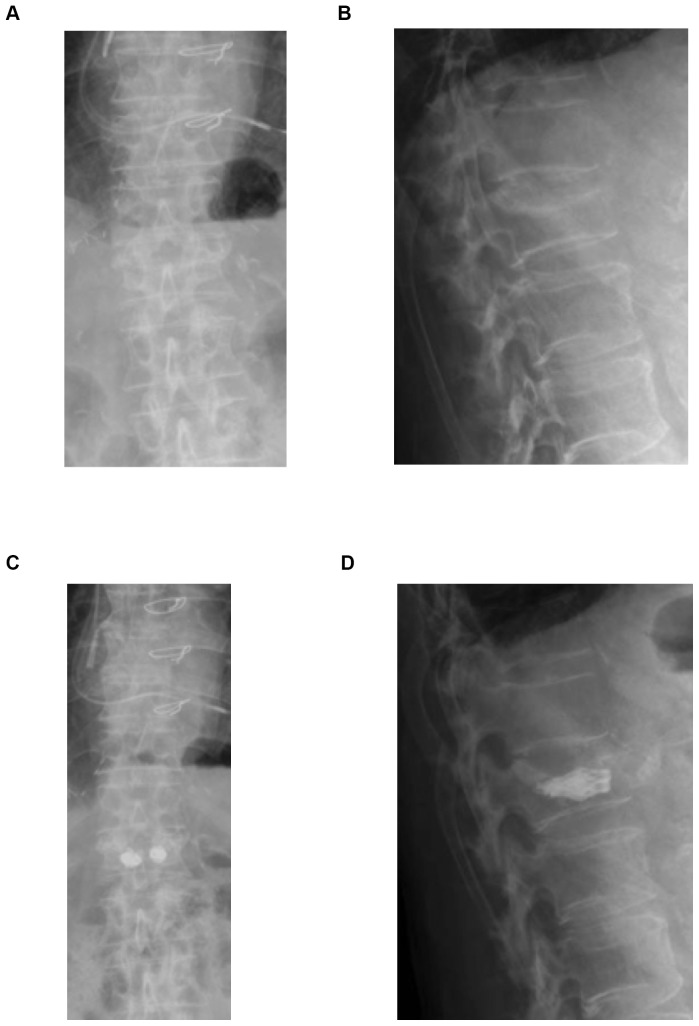
Case presentation with Osseofix® system. (a–d): Radiological case report. Preoperative (a, b) and postoperative (c, d) radiograph of L1 compression fracture and stabilization with Osseofix® system.

**Figure 4:**
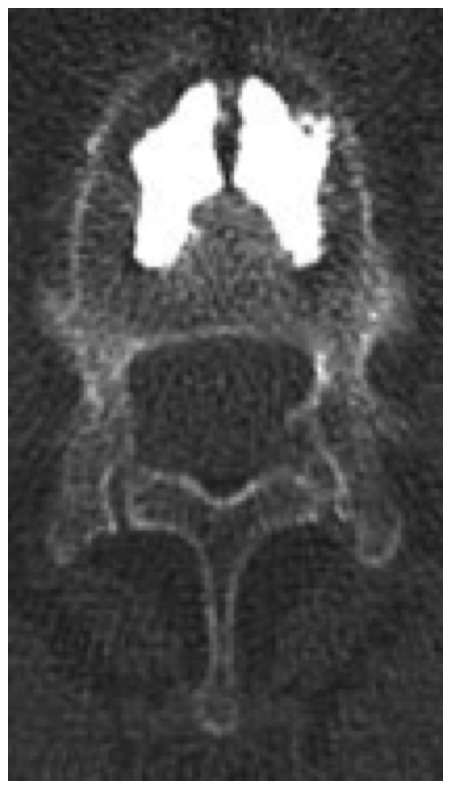
Case presentation with Osseofix® system. Radiological case report. Axial slice CT of L1 after stabilization with Osseofix® system.

Figures 3 and 4 show the radiological follow-up of a patient with an L1 fracture (VAS improvement: preoperative VAS = 8, postoperative VAS = 3, VAS at 12 months postoperative = 0).

### General complications

During the perioperative phase one complication was noticed. In one case we saw a pronounced postoperative hematoma without required treatment after a patient suffered an intraoperative hypertensive crisis with systolic blood pressures of >200 mmHg. Further postoperative complications (up to 3 months after intervention), such as neurological disturbance, hemorrhage, wound healing abnormalities, infection, phlebothrombosis, and pulmonary embolism, were not observed.

### Specific complications

In only one case at follow up we noticed a slight loss of height of the stabilized L2 vertebra. In this case the Beck index changed from 1.0 postoperative to 0.96 at follow-up. The kyphotic angle according Cobb (γ) changed from 11° to 13°. Pain scale (VAS) did not change from day of discharge (VAS = 2) to the follow-up date.

## Discussion

Kyphoplasty and vertebroplasty are well-established minimally invasive percutaneous procedures of osteoporotic thoracolumbar vertebral fractures [8] [10] [11] [13] [21]. Kyphoplasty and vertebroplasty both show good results in functional parameters as ODI in the medium term (12 months). They have also proven to produce significant pain reduction directly after the procedure as well as later on [11] [13] [22]–[26].

Vertebroplasty, which involves a short operation time and a high degree of interlock between bone cement and spongiosa, produces either a slight correction or no correction of the sagittal spine alignment [27]. Kyphoplasty, as a further development of vertebroplasty, shows partial restoration of the initial sagittal spine alignment [27] [28]. In vertebral bodies that are stabilized with vertebroplasty the primary height gain is followed by a secondary loss of height (with a certain rekyphosis as a consequence) in 18% (follow-up 6 month) to 63% of cases (follow-up 27 month) [29] [30]. After kyphoplasty loss of height was reported 12,5% and 14,3% (follow-up 12 month) to 50% (follow-up 6 month) of the cases [31]–[33]. The existing literature has little information on secondary loss of height however [34]. The most common complications associated with vertebroplasty and kyphoplasty are uncontrolled bone cement leakage over the venous plexus, which can even result in a pulmonary embolism. Leakage of the cement into the epidural space might cause neurological damage and even paraplegia [10] [13] [35]. Due to the elevated risk of cement leakage into the epidural space, in fractures that involve the posterior vertebral wall, these two procedures should only be used with great caution [22]. The incidence of cement leakage and associated complications is significantly lower for balloon kyphoplasty [13] [23] [36]–[38]. One explanation is that the cement is applied with less pressure into the previously hollowed out intraosseous space [22].

Since 2009 an interesting alternative has been available: the Osseofix® percutaneous stabilization system. This is a titanium mesh cage that also offers the possibility of minimally invasive percutaneous stabilization of the above-described spinal injuries [14] [15].

Follow-up examinations of patients treated with the Osseofix® system also showed good results. According to the Smiley-Webster scale in 23 cases (96%) we saw excellent or good clinical results. In one case the result was fair (4%). The average ODI improved from 70.6% before surgery to 30.1% at follow-up after 12 month. There was a significant reduction in pain intensity based on the VAS, with results of 7.7 preoperative, 1.7 postoperative and 1.4 at follow-up after 12 month. Our study therefore found comparable results to kyphoplasty and vertebroplasty after 12 month in the functional outcome (ODI) and pain relief (VAS).

Follow-up radiological studies, which were performed postoperative and 12 months after surgery, showed no adjacent vertebral fractures or changes in the vertebral posterior wall. Compared to the adjacent fracture rate for vertebroplasty (0–7.8%) and kyphoplasty (25–26%) (follow-up 3 to 12 month) [12] [35] [39] these results are very good. Possibly the Osseofix® systems changes the biomechanics of the vertebrae in a way that stabilization with bone cement alone (kyphoplasty) does not [14] [15]. Frequently quoted explanations for the association between vertebroplasty/kyphoplasty and increased risk of adjacent fracture are intradiscal cement leakage and straightening of the vertebral bodies [40]–[42]. We applied only very limited amounts of cement to fill the implant and therefore no cement leakage was found nor any adjacent fracture.

In only one case at follow-up we noticed a slight loss of height of the stabilized L2 vertebra. The rate of height loss found in this study at 3.1% is significantly lower compared to results after kyphoplasty or vertebroplasty. It is important to note that the Osseofix® system does not straighten the vertebral body into an upright orientation in the same way that kyphoplasty does. Lin et al. [30] found a significantly increased risk of vertebral refractures after vertebroplasty as a function of straightening of the vertebral bodies.

In our study the average vertebral kyphotic angle (α-angle) showed significant improvement when comparing the preoperative angle (9.0°) to the angle at follow-up (8.1°). The average kyphotic angle according to Cobb (γ-angle) showed significant improvement when comparing the preoperative angle (11.7°) to the angle at follow-up (10.4°). Similar changes after the same follow-up have been found for kyphotic angles following vertebroplasty [25] [43]. The results for kyphoplasty are better with an average improvement of the kyphotic angle according to Cobb of 8° [23] [25] [35] [44]–[46]. Interestingly we found no association between improved kyphotic angle according to Cobb and the clinical findings (ODI and VAS) (p = 1.0). All patients improved in ODI and VAS independently if the alignment was improved or not.

With the Osseofix® system only small amounts of bone cement are applied to an existing hollow space (expanded titanium mesh, average of 0.6 ml per mesh).

No clinically apparent cement leakages were seen in this study. A 0% leakage rage is very low compared to rates of 20–70% for vertebroplasty (3% symptomatic) and 4–13.4% (1.3% symptomatic) for kyphoplasty [28] [35] [47] [48] or 6.1% for radiofrequency kyphoplasty [49]. This is especially noteworthy in light of the association between intradiscal cement leakage and the occurrence of adjacent fractures [40] [41] [50]. Therefore the Osseofix® system seems to have an excellent safety profile. For vertebral compression fractures with posterior wall involvement the use of the Osseofix® system seems to be safe.

## Conclusion

The stabilization of symptomatic osteoporotic vertebral compression fractures with the Osseofix® system is a safe and effective procedure which reduces pain at a low rate of complications (0% cement leakage rate, 0% adjacent fracture rate, 3.1% loss of height rate). Similar to kyphoplasty, it is an easily manageable procedure and can also be used safety when the posterior vertebral wall is involved. Effects on sagittal alignment do not correlate with the clinical results. Although only limited changes in sagittal alignment (average 1–2° change of the kyphotic angle according to Cobb) was found the ODI and the VAS improved independently from the degree of kyphosis. This procedure offers an interesting alternative to the established cement augmentation procedures. The combination of less cement filled with less pressure into a mesh cage resulted in 0% cement leakage rate. The implant may be used as a cement-free implants in the future as well.
